# Cryo-EM of nucleosome core particle interactions in trans

**DOI:** 10.1038/s41598-018-25429-1

**Published:** 2018-05-04

**Authors:** Silvija Bilokapic, Mike Strauss, Mario Halic

**Affiliations:** 1 Department of Biochemistry, Gene Center, University of Munich LMU, 81377 Munich, Germany; 2 Cryo-EM facility, Max Planck for Biochemistry, 82152 Martiensried, Germany

## Abstract

Nucleosomes, the basic unit of chromatin, are repetitively spaced along DNA and regulate genome expression and maintenance. The long linear chromatin molecule is extensively condensed to fit DNA inside the nucleus. How distant nucleosomes interact to build tertiary chromatin structure remains elusive. In this study, we used cryo-EM to structurally characterize different states of long range nucleosome core particle (NCP) interactions. Our structures show that NCP pairs can adopt multiple conformations, but, commonly, two NCPs are oriented with the histone octamers facing each other. In this conformation, the dyad of both nucleosome core particles is facing the same direction, however, the NCPs are laterally shifted and tilted. The histone octamer surface and histone tails in trans NCP pairs remain accessible to regulatory proteins. The overall conformational flexibility of the NCP pair suggests that chromatin tertiary structure is dynamic and allows access of various chromatin modifying machineries to nucleosomes.

## Introduction

Packaging of DNA into chromatin regulates transcription, recombination, DNA repair and, in general, access to the genetic material. The nucleosome core particle (NCP), the fundamental repeating unit of the chromatin^1,2^, is composed of two copies of each of the histones H2A, H2B, H3 and H4 and ~150 bp of DNA wrapped around the assembled histone octamer^3^. The DNA is stably packed on the surface of the histone octamer by numerous electrostatic interactions and hydrogen bonds^3^. A short stretch of linker DNA connects the successive NCPs and is often bound by the linker histones, H1 or H5^2,4^.

The long linear nucleosome polymer is extensively condensed within an interphase chromosome. During mitosis this chromatin chain is further packaged into mitotic chromosomes. It has been suggested that the 10 nm chromosomal fiber first forms a helical 30 nm chromatin structure^5–10^, that is a necessary folding intermediate in the assembly and maintenance of condensed interphase and mitotic chromosomes. The structural and biochemical data suggested that that H4 tail of one nucleosome can bind the H2A/H2B-acidic patch of a neighboring nucleosome^5,8–11^, which might mediate folding into 30 nm fibers.

Many recent results, however, show that no defined structures beyond the 10 nm fiber were present in the chromatin of interphase or mitotic chromosomes, even in the highly condensed heterochromatic regions^12–20^. Super resolution imaging revealed that nucleosomes form more heterogeneous, and not well-defined assemblies *in vivo*^21^. These assemblies are dynamic and are organized by cohesin and nucleosome-nucleosome interactions^19^. Recent ChromEMT data show that within the cell nucleosomes are assembled into disordered 5- to 24-nm-diameter chains with different nucleosome arrangements, densities and conformations^20^. These data indicate that defined 30 nm structure might not be prevalent structure *in vivo* and that chromosomes are assembled through long range interactions of extended 10 nm fibers which form an interdigitated polymer-like structure.

Chromosome conformation capture experiments suggest that interphase chromosomes are organized into globular structures called topologically associating domains (TADs)^22–25^. These data suggest that the long range interactions of the 10 nm chromatin fiber are important determinants of the structure and organization of chromatin. Despite its importance, relatively little is known about the long range interaction of nucleosomes. In this work, we solved cryo-EM structures of differently interacting nucleosome core particles revealing long range nucleosome interactions that we suspect may drive chromatin compaction. Our structures show that the mono-NCP pairs can adopt many conformations, but are commonly oriented with histone octamers facing each other. The overall conformational flexibility of NCP pairs suggests that chromatin tertiary structure is dynamic and allows access of various chromatin modifying machineries to nucleosomes.

## Results

### Cryo-EM structure of nucleosome core particle pairs

**Figure 1:**
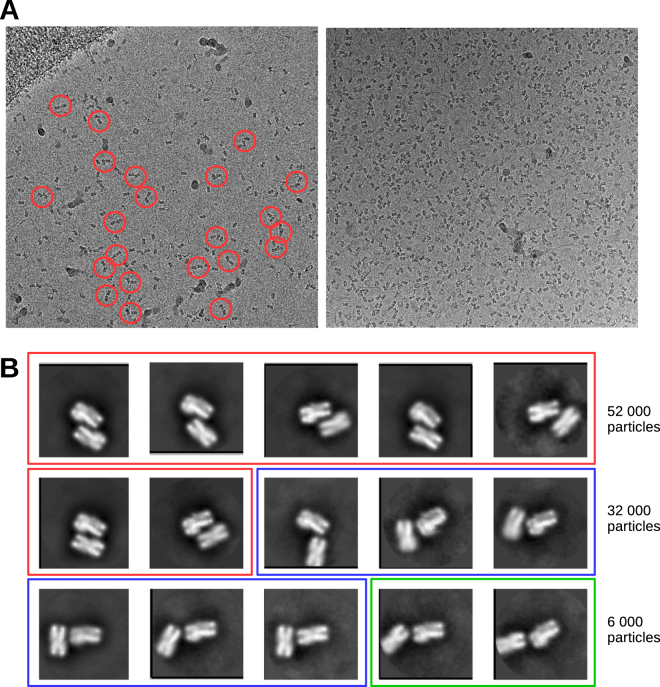
Nucleosome core particle dimers. (**A**) Representative cryo-EM raw micrographs collected on a Falcon II camera with a Titan Halo electron microscope at 300 keV. Many NCP pairs are also visible. Several NCP pairs are depicted by red circles. (**B**) Representative 2D class averages showing pairs of nucleosome core particles in many different orientations. Red area: particles with two histone octamers facing each other. Blue area: particles with the histone octamer facing the DNA of the second NCP. Green area: particles with the two DNA regions of the NCPs facing each. In nearly all cases, the gyre of the DNA around the histone core is visible.

We collected cryo-EM data of nucleosome core particles assembled on a 149 bp long 601 DNA sequence at physiological salt conditions (150 mM NaCl)^26,27^. In the electron micrographs, NCPs are present in various orientations and often form dimers and sometimes even trimers (Fig. 1A, red circles). Even at the low particle density, nucleosome core particle pairs are commonly present (Fig. 1A, left panel).

Of 574 000 NCP particles analyzed, 2D classification revealed that 108 000 particles had a second NCP in the proximity. This comprises ~20% of all particles. On the images with low particle density 31 000 NCPs out of 121 000 had a second particle in the proximity, indicating that particle density is not a major determinant of inter-NCP interactions. We performed additional 2D classification of these particles and observed that NCPs can arrange in many ways (Figs 1B and S1C). The most common arrangement of two NCPs in the pair was side-by-side packing with two histone octamers facing each other (Fig. 1B). This packing is related to the nucleosome packing in the putative 30 nm fiber and tetranucleosome structure^5,9,28^ and approximately 52 000 NCP (~50%) particles interact in this way (Fig. 1B, red area). However, in contrast to defined interactions in 30 nm fiber, we observe many possible conformations of NCP pairs (Fig. 1B). In addition to side-by-side packing, more than 30 000 (~28%) NCPs were arranged in a perpendicular orientation, in which DNA of the first NCP interacts with the histone octamer or DNA of the second NCP (Fig. 1B, blue area). This perpendicular orientation might represent initial contact which could lead to a more stable side by side interaction. Approximately 6000 (~6%) NCP pairs were oriented such that their DNA was apposed (Fig. 1B, green area). The remaining ~16% of the particles were in various other orientations (Fig. S1C).

Using all particles, we solved the structure of the NCP to 4.7 Å resolution (Fig. S2A,B). Further classification of nucleosome core particles revealed three major classes that show appearance of the second NCP. In these classes, the primary NCP (NCP 1) is resolved at ~7 Å with histone core α-helices clearly visible (Fig. S2C). Each class contained approximately 1/3 of the particles that interact with the second NCP. For these comparisons, we fixed the orientation of the first NCP (NCP 1) and looked at the position of the second NCP (NCP 2) in relation to NCP 1.

In the first class (Class A), the density for the second NCP is parallel to the histone octamer core of NCP 1 (Fig. S2D). In the Class B, the density for the second NCP is also on the histone octamer side but more laterally shifted and also tilted (Fig. S2D). The position of the NCP 2 in the Class A and B cryo-EM maps reflects the NCP orientations in the 2D averages that are labeled red in Fig. 1B. In Class C, the density for the NCP 2 is on top of NCP 1 (Fig. S2D) and this class is represented by the 2D class averages marked with blue in Fig. 1B. In this class, NCP 2 is in close proximity to the DNA of NCP 1. In all classes the density of the NCP 2 was very undefined indicating presence of multiple conformations (Fig. S2D).

**Figure 2:**
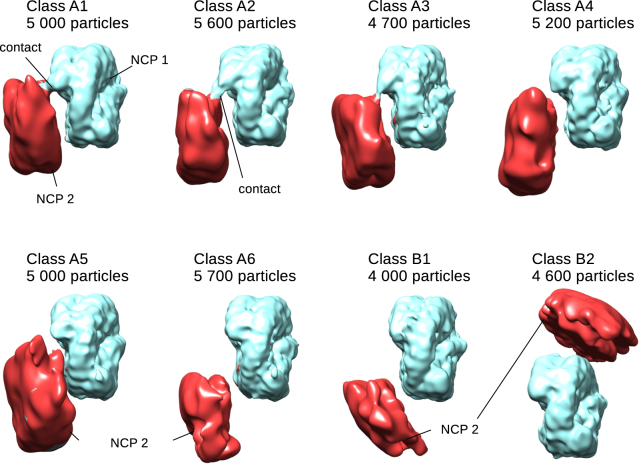
NCP pairs can adopt multiple conformations. Cryo-EM maps of the classes of NCP pairs showing their diverse relative orientations. NCP 1, for which the orientation is fixed between classes, is shown in light blue, and NCP 2 is shown in red. The number of particles for each class is indicated. Note that the strongest contact observed in classes A1–A3 is formed proximal to the entry/exit site of NCP 1 and the DNA of NCP 2.

We further classified each of classes A, B and C and obtained 8 classes with the distinct density for NCP 2 (Fig. 2). The resolution of the primary NCP 1 in these classes is between 8 Å and 10 Å, while NCP 2 is present at much lower resolution of 15–25 Å (Fig. S3). In five of the classes (A1–A5), NCP 2 is arranged parallel to NCP 1, with two histone octamers facing each other, but offset vertically such that histones on each could be contacted (Fig. 2). In classes A6 and B1, NCP 2 is on the octamer side of the NCP 1, but laterally shifted and tilted (Fig. 2). In this conformation the DNA of the two NCPs is in the close proximity. In the Class B2, NCP 2 is found on top of NCP 1 (Fig. 2). In this conformation the histone octamer of the NCP 2 faces the DNA of NCP 1. For Class C we did not obtain a map with a defined density for NCP 2 leading us to conclude these are transient or poorly ordered occurrences.

### The trans-interaction between two nucleosome core particles

**Figure 3:**
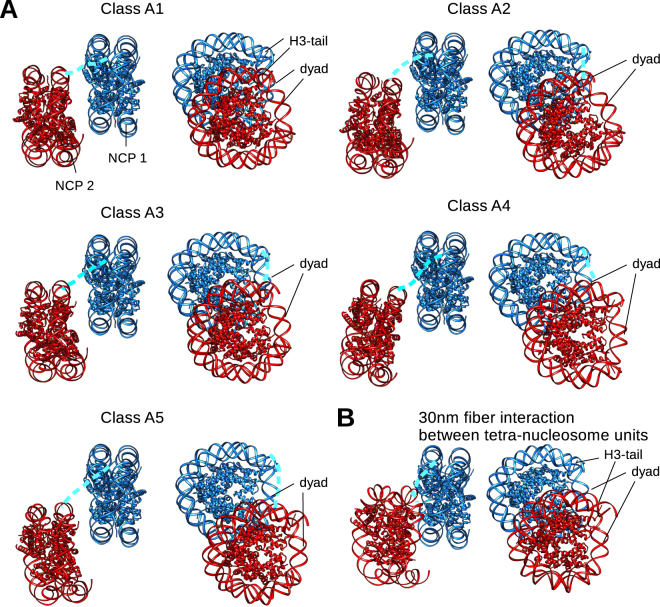
Molecular model showing long range NCP interactions. (**A**) Comparison of the NCP models for cryo-EM maps A1–A5. NCP 1 is shown in blue and NCP 2 is shown in red. The two NCPs are oriented with their histone octamers facing each other, but are shifted vertically to different degrees. The dyad of both NCPs is facing in the same direction. First 37 residues of the H3-tail, not observed in X-ray structure (pdb 3lz1), are indicated as a dotted line in light blue. In most classes, the histone octamer surface remains accessible to the extrinsic factors. (**B**) The model showing NCP orientation in the 30 nm fiber between adjacent tetra-nucleosomal units is included for comparison. The 30 nm fiber model was built using 30 nm fiber cryo-EM map^5^.

In Classes A1–A3 we observe a strong contact between NCP 1 and NCP 2 (Fig. 2). In classes A4 and A5, the two nucleosome core particles are farther apart and the contact between them appears weaker (Fig. 2). At lower contour levels, however, the contact between two NCPs can be observed as well (Fig. S4). We fitted the crystal structure of the nucleosome core particle (PDB:3LZ1) into both NCPs of the pair. For NCP 2 we could reliably fit the crystal structure into classes A1–A5 (Fig. S5). In classes A6, B1 and B2 the density for the second NCP is not sufficiently defined to unambiguously orient the X-ray model. We observed that in all classes both NCPs are parallel to one another with the dyad facing the same direction (Fig. S5). We compared the NCP orientations in our structures with nucleosome stacking in the published model of the 30 nm fiber^5^. Within the tetra-nucleosome unit in the 30 nm fiber, two NCPs are stacked parallel and at the nearly same height (Fig. S6A). This leads to strong contacts between two NCPs, mediated by H2A/H2B four helix bundles between two neighboring nucleosomes (Fig. S6A,B). In all of our structures, however, NCP 2 is vertically shifted between 30–70 Å relative to NCP 1 (Figs 2 and 3). This mode of interaction bears a greater similarity to the weaker interaction between NCPs belonging to two different tetra-nucleosome units in the 30 nm fiber which are mediated by the N-terminal tail of histone H4 of one nucleosome and the “acidic patch” on the H2A/H2B faces of the opposed nucleosome^5^ (Figs 2 and 3).

Since our NCPs are not artificially forced into the 30 nm fiber, we observe many possible arrangements of the nucleosome core particles in pairs. In most of our classes we observe a contact between the entry/exit site of NCP 1 and the DNA of NCP 2. Since this contact spans long distance it might be mediated by the N-terminal tail of the histone H3 and the DNA. H3 tail exits histone core at the place of the contact and biochemical characterization of nucleosome interactions has shown that the H3 tail is required for interactions between two 10 nm fibers^29–31^. It has been also shown by ChIP-exo that H3 tail interacts with DNA *in vivo*^32^.

**Figure 4:**
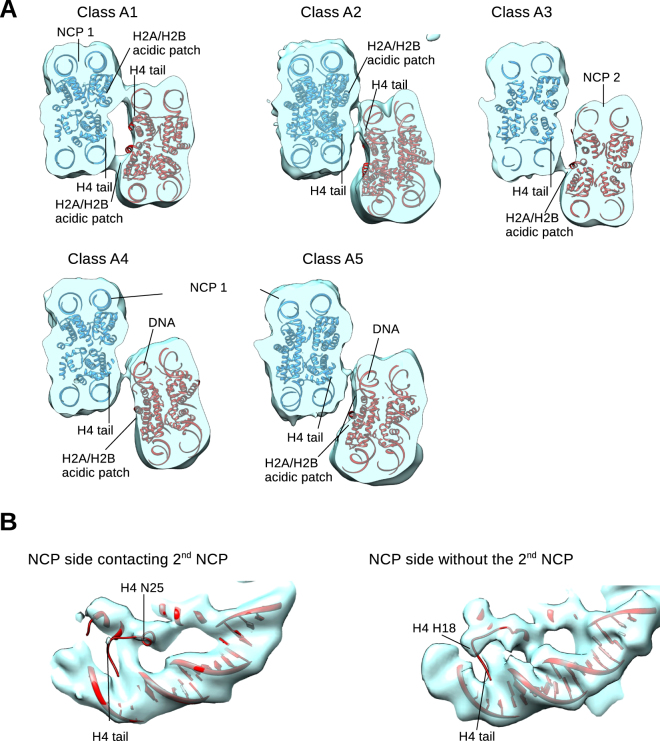
H4 tail makes weak interactions with H2A/H2B or DNA. (**A**) A slice through the NCP pairs of classes A1-A5 showing weaker interactions at the histone octamer interface. These contacts are presumably formed by the histone H4 tail of one NCP and the H2A/H2B acidic patch or the DNA of the second NCP. The cryo-EM density for both NCPs is shown in transparent blue. The molecular model for NCP 1 is shown in blue and for NCP 2 in red. (**B**) The cryo-EM map of the combined reconstruction of the classes A1–A3. On the histone octamer side without the adjacent NCP 2, the strong density where the N-terminus of H4 interacts with DNA at superhelix SHL 2 is visible. On the opposite side, with the adjacent NCP 2, the density for the H4 tail is not visible. This indicates that the H4 tail dissociates from the DNA of the NCP 1 and engages in intra-nucleosomal interactions.

We also observe weaker contacts between two histone octamers, that are visible at lower contour levels (Fig. 4). These contacts might be mediated by the N-terminal tail of the histone H4 and the acidic patch of H2A/H2B which form primarily contact in the *in vitro* assembled 30 nm fiber and have been observed in several crystal structures^5,33,34^. In the Class A1, the two NCPs are almost at the same height and this interaction is most similar to the interaction observed in the 30 nm fiber (Figs 4A and S6A). In this class we observe two contacts on the octamer side that likely involve the acidic patch of one NCP and the H4 tail of the second NCP. In Class A2, NCP 2 is displaced laterally and the interaction at the octamer surface is weaker. The acidic patch and the H4 tail are no longer aligned, leading to weaker interactions. We do, however, still observe two weak contacts in this conformation. In Class A3, NCP 2 is further shifted and tilted in-plane and we observe only one weak contact between the acidic patch of NCP 2 and possibly H4 tail of NCP 1. In Classes A4 and A5, the vertical offset of NCP 2 is even more pronounced and the H4 tail of NCP 1 cannot reach the acidic patch of NCP 2. In these classes we only observe very weak contacts on the octamer side between NCP 1 and 2, possibly between the H4 tail and DNA (Fig. 4A).

Next, to obtain better resolution, we combined particles of classes A1-3, which show the strongest interaction between NCP 1 and NCP 2 at the octamer side. In the combined reconstruction NCP 1 is resolved to 8 Å and we can observe the density for the H4 tail until histone H4 ~His18 and at lower contour level to histone H4 ~Ala15 on the side where there is no NCP 2 bound. On the side that interacts with the NCP 2, the H4 tail does not bind the DNA anymore (Figs 4B and S6B–E). The last residue of the H4 tail that is enclosed by the cryo-EM density is H4 Asn25. This indicates that the basic patch of H4 (Arg16-Arg23) is involved in the interaction with the second nucleosome. Our data show that the H4 tail interacts with the DNA of its own NCP, and in the presence of the second NCP it dissociates from the own DNA and engages in the inter-NCP interactions (Figs 4B and S6E). The density for the H4 tail interacting with H2A/H2B of the neighboring nucleosome, was also observed in cryo-EM structure of the 30 nm fiber and multiple X-ray structures^3,5,9,28^. The interactions observed in our cryo-EM structures allow flexibility in the nucleosome core particles arrangements, which might be essential to permit access to many factors that bind nucleosomes at the histone octamer surface^34–42^.

## Discussion

Although it was suggested that chromatin folds into the 30 nm fiber, recent data indicate that defined 30 nm structure might not exist *in vivo*. The latest results show that nucleosomes form a less defined interdigitated polymer like structure in which nucleosomes have many different arrangements and conformations^16,19–21^. How two distant nucleosomes interact in this polymer-like structure is not well understood. Using cryo-EM we have solved several structures showing interactions of two nucleosome core particles that are not directly linked by their DNA. Our structures reveal the intrinsic interactions between two nucleosome core particles that might be relevant for the long range nucleosome packing between different 10 nm fibers.

Consistent with the biochemical and structural results^5,29–31,43–45^, our data suggest that the H3 and H4 tails make essential contacts between two NCPs in the nucleosome pair. In our structures the main contact between two NCPs is near the DNA entry exit/site, which suggests that the long H3 tail makes the initial contact with the DNA of the second NCP. Biochemical and small-angle X-ray scattering data have shown that the H3 tail is extensively involved in inter-nucleosomal interaction and engages in inter-array interactions^29,46^. This would tether the second nucleosome close to the first nucleosome leading to secondary contacts between the H4 tail and the acidic patch or DNA^5,45,47^. In Classes A4 and A5, the two NCPs are too distant for the interaction between the basic patch of H4 and the acidic patch of H2A/H2B. In these classes we observe that the H4 tail binds in the proximity of the DNA of the second NCP. H4 interaction with the DNA was observed by cross-linking experiments and in silico, and it has been suggested that the H4 tail can also mediate long-range inter-array interactions that stabilize tertiary chromatin structures^43,47,48^.

Our structures reveal long-range nucleosome interactions and show high conformational flexibility of the nucleosomal pair. This is consistent with the latest results showing that nucleosomes might not form a very defined 30 nm structure, but long range interactions of nucleosomes will lead to formation of less defined interdigitated polymer structure^18–21^. The overall conformational flexibility of the nucleosome pair also indicates that the histone octamer surface and the histone tails remain accessible to many regulatory proteins. This is consistent with the FRET measurements of nucleosome arrays^49^ and *in vivo* data showing that local nucleosome dynamics drive chromatin accessibility^19,50^, which is essential to regulate transcription, replication and DNA repair. Our structures indicate that NCP interactions are highly dynamic allowing access to a wide variety of chromatin modifying machineries.

## Experimental procedures

### Nucleosome core particle reconstitution

*Xenopus laevis* histones were co-expressed and co-purified as soluble H2A/H2B histone dimers and (H3/H4)2 histone tetramers, as described^26,51,52^. The purified histone pairs were used to assemble histone octamer in 25 mM HEPES/NaOH pH 7.5, 2 mM NaCl, 1 mM DTT. To obtain the histone octamer a 2.8 fold excess of wild type H2A/H2B histone dimer was mixed with H3/H4 histone tetramer. The access of H2A/H2B was purified from the histone octamer by the size exclusion chromatography equilibrated in 25 mM HEPES/NaOH pH 7.5, 2 M NaCl, 1 mM DTT and subsequently purified by size exclusion chromatography (Fig. S1A). DNA for NCP reconstitution was PCR amplified from a plasmid containing the Widom 601 DNA sequence. The PCR product was purified by phenol-chloroform extraction. After ethanol precipitation, the DNA was resuspended in 25 mM HEPES/NaOH pH 7.5, 2 M NaCl, 1 mM DTT.

The histone octamer peak fractions after size exclusion chromatography were mixed with DNA and placed into a dialysis button made from the lid of an Eppendorf tube. The NCP reconstitution was done by ‘double bag’ dialysis^26,52^. The dialysis buttons, containing 0.25 ml of the histone octamer:DNA mixture, were placed inside a dialysis bag, filled with ~50 ml of size exclusion buffer. The dialysis bag was immersed into a 1l of buffer containing 15 mM HEPES/NaOH pH 7.5, 1M NaCl, 1 mM DTT and dialysesd over-night at +4 °C. The next day the dialysis buffer was replaced with 1 l low salt buffer (15 mM HEPES/NaOH pH 7.5, 150 mM NaCl, 1 mM DTT). The dialysis into low salt buffer was done for 5–6 hours. Finally dialysis buttons were removed from the dialysis bag and dialysed for 1–2 hours into a fresh low salt buffer. The samples were concentrated for cryo-EM grids preparation to 2 mg/ml. The reconstitution results were analysed on 6% native PAGE (Fig. S1B).

### CryoEM grid preparation and data collection

Quantifoil R2/1 or R1.2/1.3 holey carbon grids were used. A Leica EM GP automatic plunge freezer was used for the sample vitrification. Temperature in the chamber was kept at +15 °C and humidity at 95%. 3 μl of NCP sample was applied to freshly glow-discharged grid. After 3 s of blotting time, grids were plunge-frozen in the liquid ethane. Electron micrographs were recorded on a FEI Titan Halo (FEI) at 300 kV with a Falcon 2 direct electron detector (FEI) at nominal magnification of 75 000x resulting in an image pixel size of 1.4 Å per pixel on the object scale. Data were collected in a defocus range of 10 000–40 000 Å with a total exposure of 100 e/Å^2^. 40 frames were collected and aligned with the Unblur software package with a dose filter^53^. We collected ~2000 micrographs from several independent NCP preparations. Several thousand particles were manually picked and carefully cleaned in XMIPP^54^ to remove inconsistent particles. The resulting useful particles were then used for semi-automatic and automatic particle picking in XMIPP. The contrast transfer function parameters were determined by CTFFIND4^55^. The 2D class averages were generated with the Relion software package^56^. Bad class averages were removed from further data analysis. The 3D refinements and classifications were subsequently done in Relion. All final refinements were done in Relion using the auto refine option. The initial reference was filtered to 60 Å in Relion. Particles were split into 2 datasets and refined independently and the resolution was determined using the 0.143 cut-off (Relion auto refine option). Local resolution was determined with Relion 2.0. All maps were filtered to local resolution using Relion 2.0 with B-factor determined by Relion (~−200). PDB 3LZ1 was fitted into cryo-EM maps with Chimera software package. Visualization of all cryo-EM maps was done with the Chimera software package^57^.

### Data availability

EM densities have been deposited in the Electron Microscopy Data Bank under accession codes EMD-4221 (Class A1), EMD-4222 (Class A2), EMD-4223 (Class A3), EMD-4224 (Class A4), EMD-4226 (Class A5), EMD-4227 (Class A6), EMD-4228 (Class B1), EMD-4229 (Class B2). All other data are available from the corresponding author upon reasonable request.

## Electronic supplementary material

Supplementary Information
