# Supplementary material for: Cryo-EM of nucleosome core particle interactions in trans
Source: Sci Rep. 2018 May 4;8:7046. doi: 10.1038/s41598-018-25429-1 (PMC5935684; doi:10.1038/s41598-018-25429-1)
Supplement: Supplementary file 1 — Supplementary Information [file 41598_2018_25429_MOESM1_ESM.pdf]

Supplementary Material

Cryo-EM of NCP interactions in trans

Authors: Silvija Bilokapic[1], Mike Strauss[2] and Mario Halic[1,*]

Affiliations:

[1]Department of Biochemistry, Gene Center,

University of Munich LMU, 81377 Munich, Germany

[2]Cryo-EM facility, Max Planck for Biochemistry,

82152 Martiensried, Germany

*Corresponding author, e-mail: halic@genzentrum.lmu.de

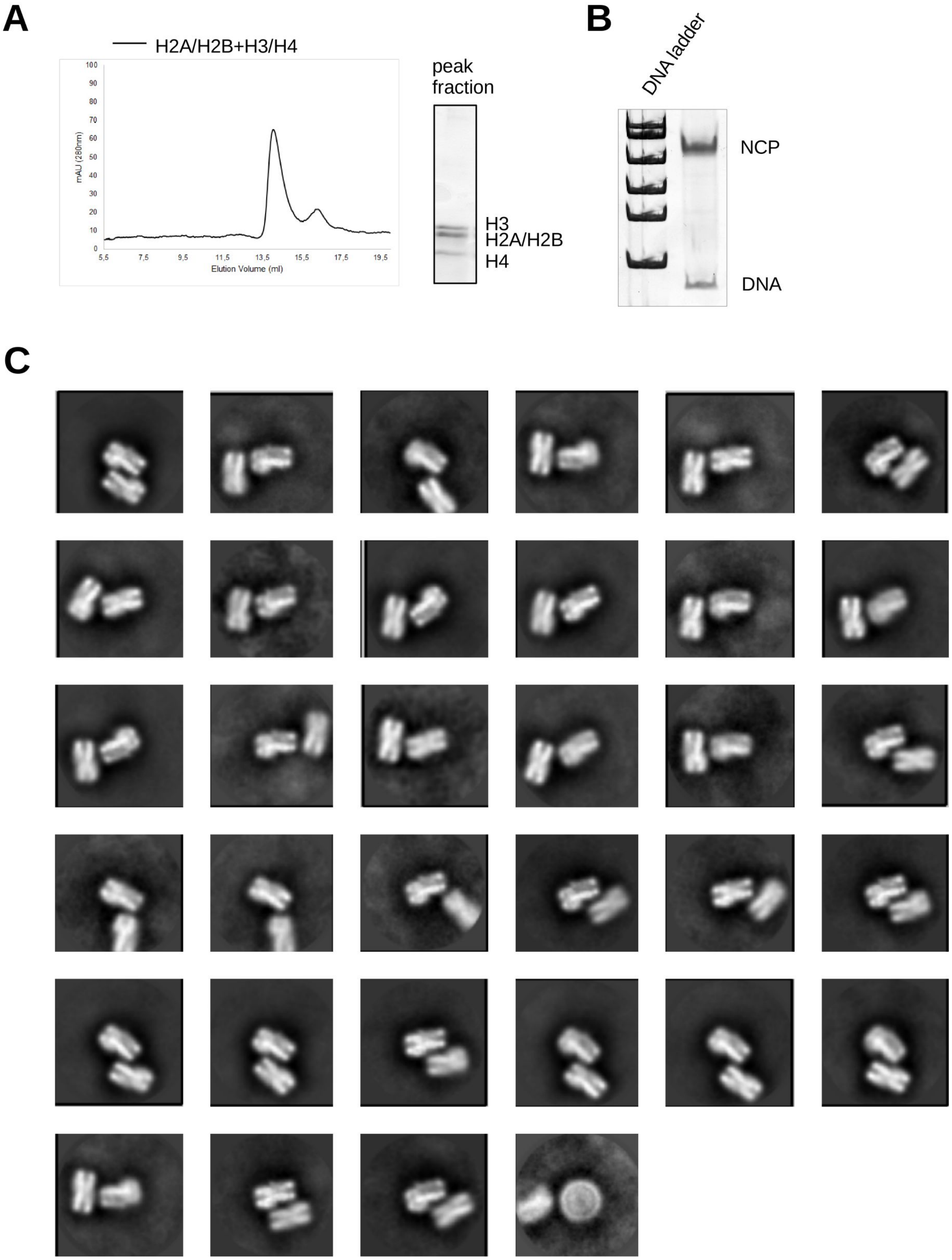

Supplementary Figure 1

Figure S1: Nucleosome core particle dimers

(A) Chromatogram and SDS-PAGE showing assembly of the native octamer. Excess of H2A/H2B dimer, used in the octamer assembly, was removed by size exclussion chromatography. Black line shows migration of the octamer. Red line shows migration of the H2A/H2B dimer. Selected fractions, marked with a black bar on the chromatogram, were analyzed by SDS-PAGE.

(B) Native gel showing NCP assembly.

(C) Representative 2D class averages showing pairs of nucleosome core particles in many different orientations. Many details are visible in 2D class averages.

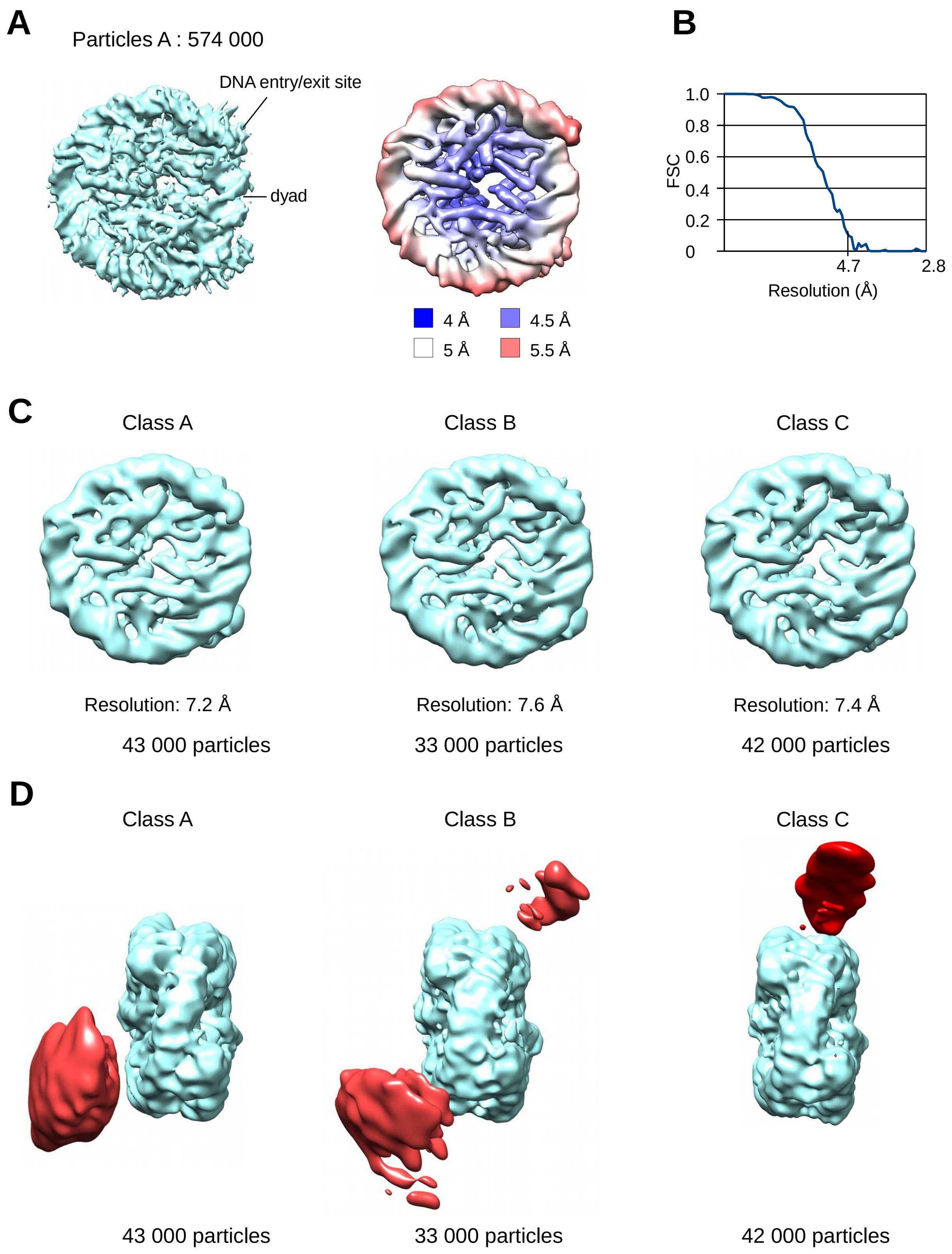

Supplementary Figure 2

Figure S2: Cryo-EM of NCP dimer particles

(A) Cryo-EM map and local resolution estimation of the NCP at 4.7 Å (0.143 cutoff in FSC curve).

(B) Fourier shell correlation (FSC) curve showing the resolution of cryo-EM map of the NCP.

(C) Cryo-EM maps of NCP 1 of the 3 classes that have the adjacent NCP 2.

(D) A lower counter of the 3 classes that have the adjacent NCP 2. The density for NCP 2 is shown in red. NCP 1 is shown in blue.

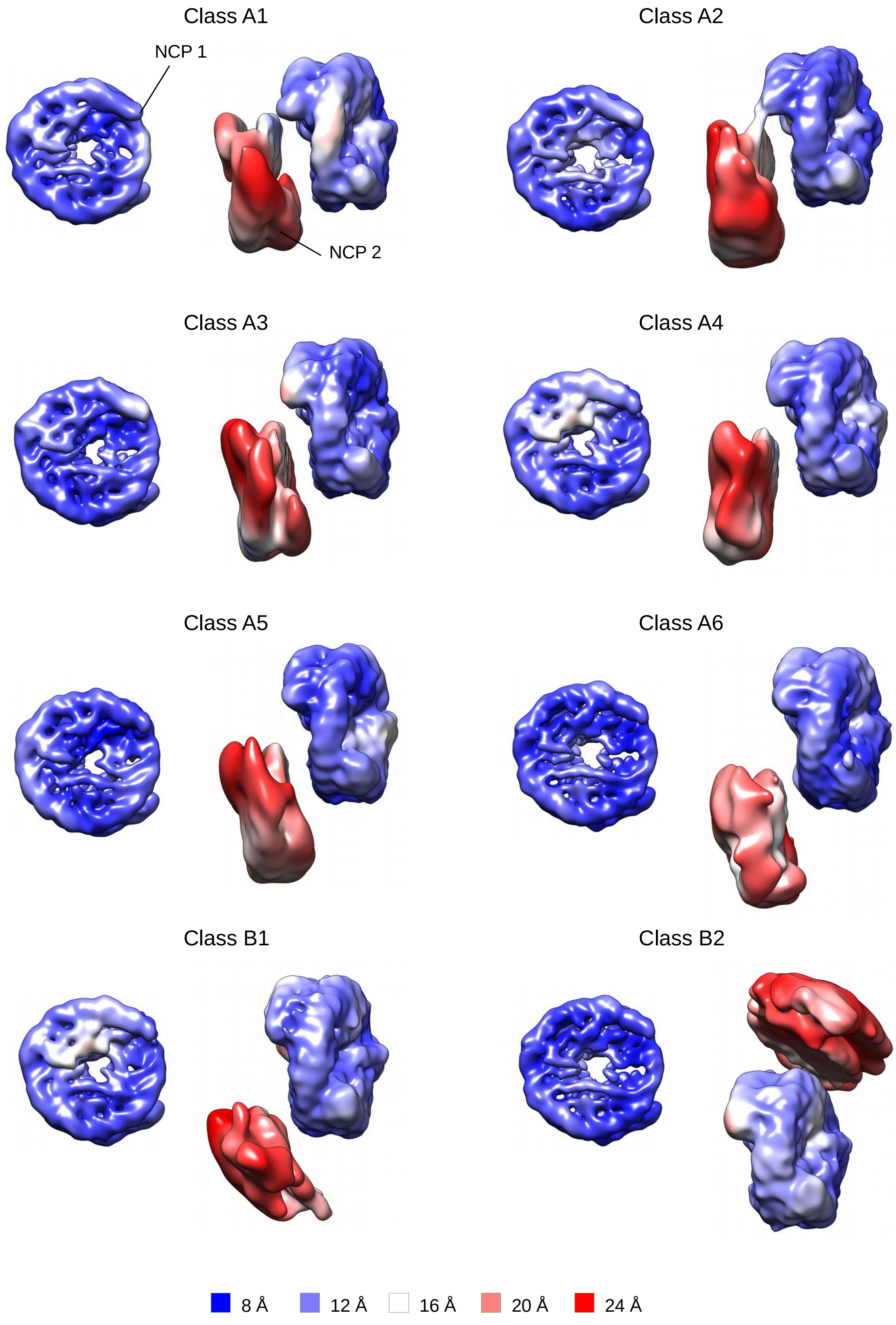

Supplementary Figure 3

Figure S3: Local resolution calculation of NCP dimer reconstructions

Local resolution estimate determined by Relion. NCP 1 is resolved at 8-10 Å, while the NCP 2 is present at much lower resolution (15-25 Å). The side view is shown at higher contour level to show local resolution of NCP 1.

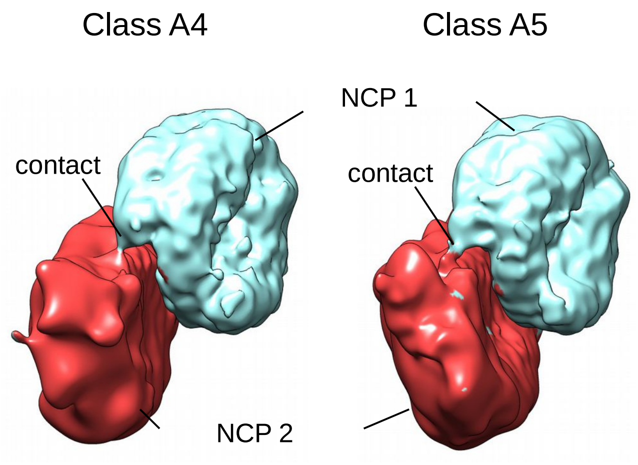

Supplementary Figure 4

Figure S4: NCP pairs can adopt multiple conformations

(A) Lower contour level of cryo-EM maps A4 and A5 showing the contact NCP 1 and NCP 2. The NCP 1 is shown in light blue and the NCP 2 is shown in red.

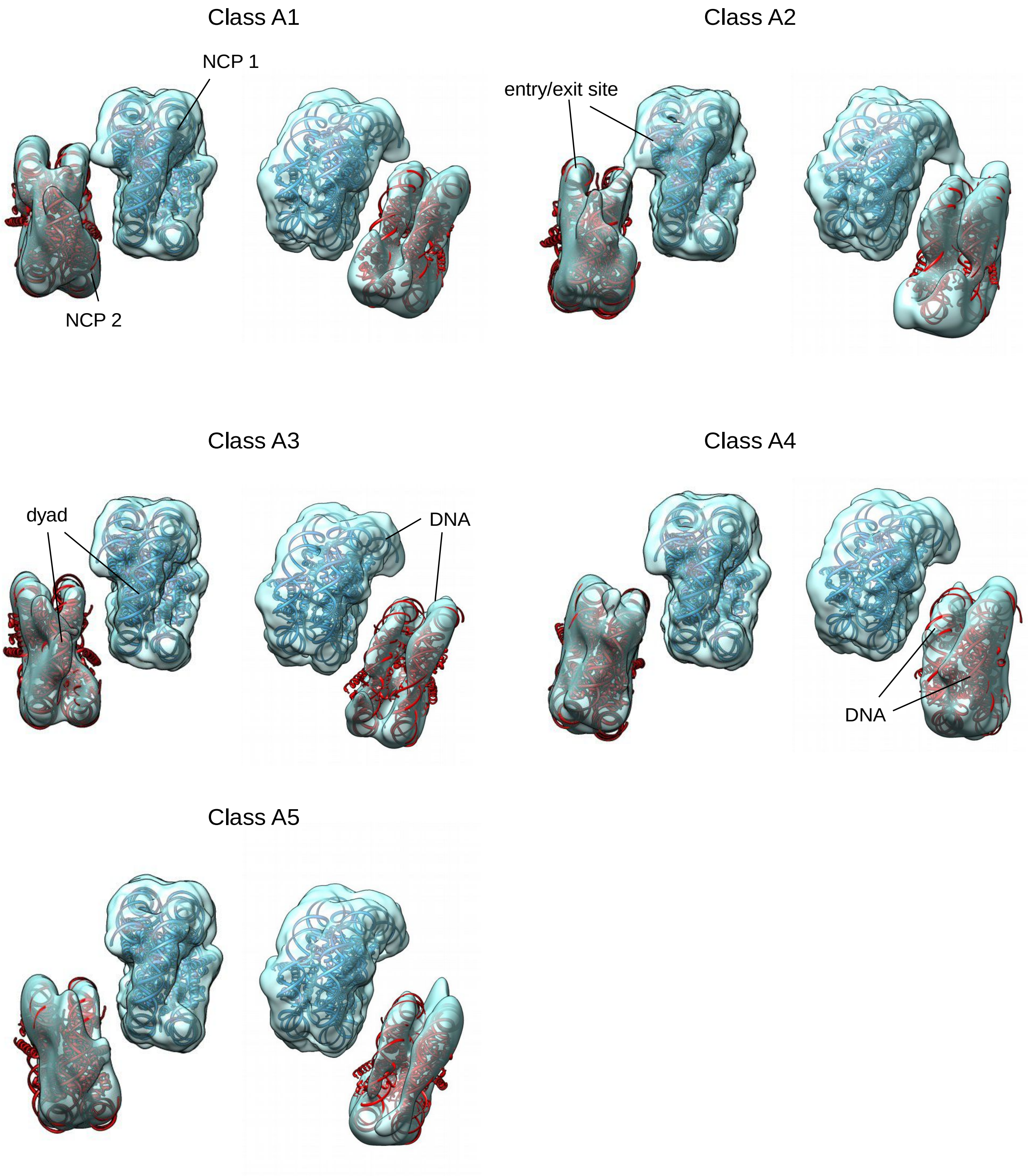

Supplementary Figure 5

Figure S5: Fitting of the X-ray model into cryo-EM maps.

The X-ray model of the NCP (PDB ID 3LZ1) was fitted into the Classes A1-A5. Cryo-EM density for both NCPs is shown in transparent blue. The molecular model for NCP 1 is shown in blue and for NCP 2 in red.

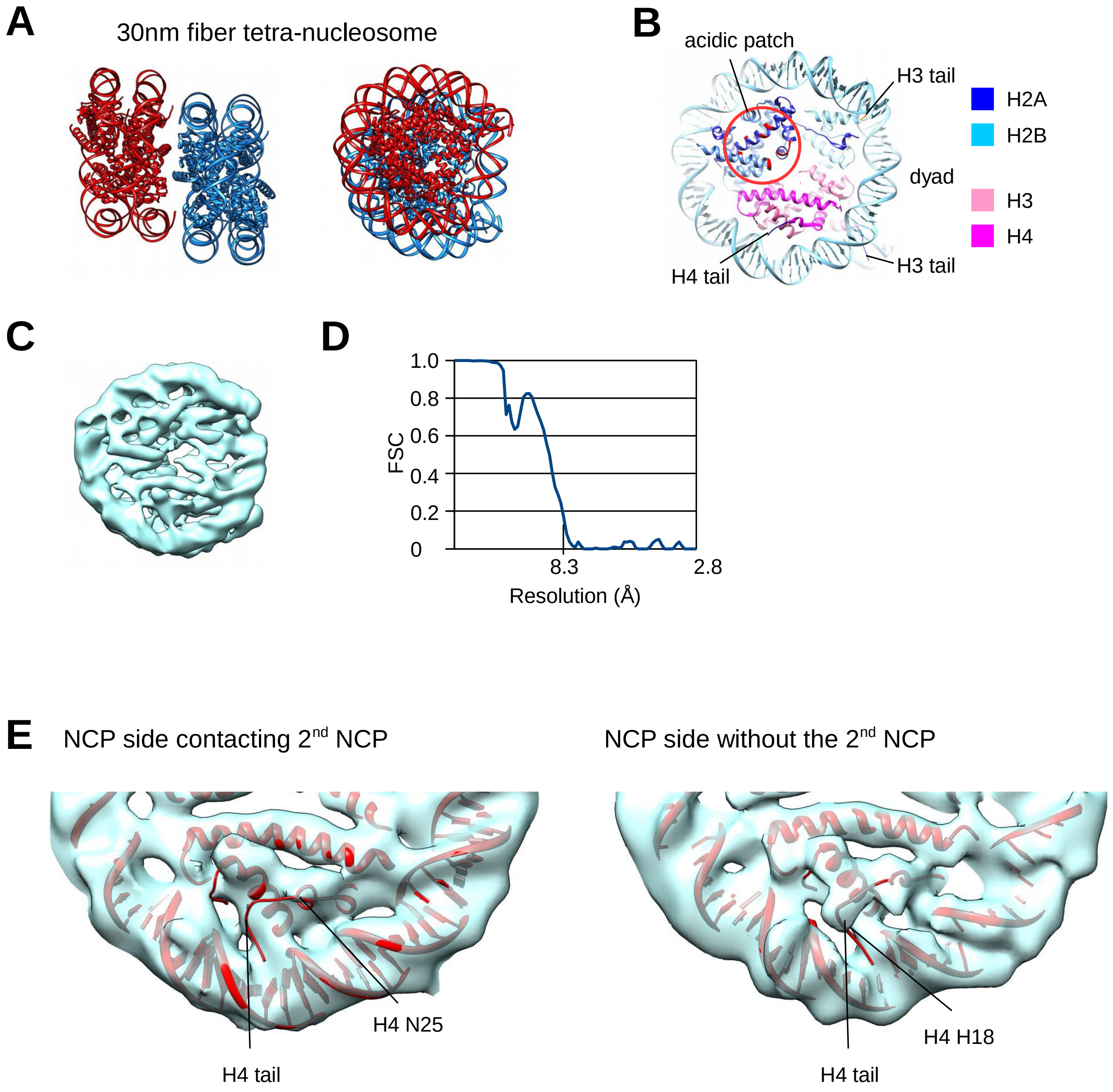

Supplementary Figure 6

Figure S6: H4-tail delocalizes when adjacent to the second nucleosome core particle

(A) Comparison of the NCP models fitted into the tetra-nucleosome structure of 30 nm fiber (Song *et al*, 2014). In the tetra-nucleosome, adjacent NCPs are stacked with histone octamers facing each others.

(B) NCP model with depicted histones, histone tails and acidic patch (red circle).

(C) Cryo-EM map of the combined reconstruction of the classes A1-A3 at 8.3 Å (0.143 cutoff in FSC curve).

(D) Fourier shell correlation (FSC) curve showing the resolution of cryo-EM map of the NCP.

(E) H4 tail is changing the conformation upon the interactions with the second NCP.

References :

Song F, Chen P, Sun D, Wang M, Dong L, Liang D, Xu R-M, Zhu P & Li G (2014) Cryo-EM study of the chromatin fiber reveals a double helix twisted by tetranucleosomal units. *Science* 344: 376–380
